# Deep neck infection - analysis of 80 cases

**DOI:** 10.1016/S1808-8694(15)31097-1

**Published:** 2015-10-19

**Authors:** Alexandre Babá Suehara, Antonio José Gonçalves, Fernando Antonio Maria Claret Alcadipani, Norberto Kodi Kavabata, Marcelo Benedito Menezes

**Affiliations:** 1 Head and neck surgeon, assistant in the Head & Neck Surgery Discipline, Sao Paulo Santa Casa; 2 Adjunct professor, chief of the Head & Neck Surgery Discipline, São Paulo Santa Casa; 3 Doctoral degree, assistant in the Head & Neck Surgery Discipline, São Paulo Santa Casa; 4 Master's degree, assistant in the Head & Neck Surgery Discipline, São Paulo Santa Casa; 5 Doctoral degree, assistant in the Head & Neck Surgery Discipline, São Paulo Santa Casa. Medical Science School, Sao Paulo Santa Casa, Head & Neck Surgery Discipline

**Keywords:** guideline, complications, diagnosis, infection, neck, treatment

## Abstract

Deep neck infections are serious diseases that involve several spaces in the neck. The most dreadful complication is descending necrotizing fasciitis, which needs early diagnosis and aggressive treatment.

**Aim:**

To analyze 80 treated cases of deep neck infection and propose a schematic guideline for managing this disease.

**Method:**

The authors present a retrospective analysis of 80 treated cases of deep neck infection, from June 1997 to June 2003.

**Results:**

Odontogenic and tonsilar causes were the more frequent ones. Submandibular and parapharyngeal spaces were the most frequent location of deep neck infection. Staphylococcus aureus and Streptococcus sp were the microorganisms more commonly isolated.

**Conclusions:**

Airway control should be priority in managing deep neck infections and if the patient has to be submitted to surgery special care should be taken at the moment of intubation - when curare must never be used. CT scan is the gold-standard imaging evaluation for the diagnosis of deep neck infection. Morbi-mortality is high when associated with septic shock and mediastinitis. Our mortality rate was 11.2% and only one, in five patients with mediastinitis, survived.

## INTRODUCTION

Deep neck infections (DNI) are bacterial infections originating from the upper aerodigestive tract and involving the deep neck spaces. Although uncommon, these infections are severe, and if not treated adequately, may lead to death. The incidence of this disease was relatively high before the advent of antibiotics, requiring prompt recognition and early interventions.^1^ Antibiotics resulted in a significant decrease in the occurrence and the progression of this disease. However, when not diagnosed and treated appropriately, these infections progress rapidly and are associated with high morbidity and mortality.

Descending necrotizing mediastinitis is the most feared complication; it results from retropharyngeal extension of infection into the posterior mediastinum. Septic shock is associated with a 40–50% mortality rate.^2^^,^^3^ Pleural and pericardic effusion may accompany this condition, frequently leading to cardiac tamponade. Furthermore, suppurative thrombophlebitis of the internal jugular vein associated with pulmonary septic embolism, thrombosis of the cavernous sinus and erosion of the carotid artery have been reported.^4^

The purpose of this paper was to retrospectively analyze 80 cases of deep neck infection treated between June 1997 and June 2003. An algorithm for managing this condition is proposed.

## MATERIAL AND METHOD

The study was a retrospective analysis of 80 treated cases admitted into the emergency unit between June 1997 and June 2003.

Data collection involved demography (age, sex and race), social habits (smoking and consumption of alcoholic beverages), and information on associated diseases.

Also studied were the clinical presentation of the disease, the duration of hospital stay, laboratory exams, the etiology, bacteriological studies, the treatment and the complications.

Computed tomography of the neck and thorax and surgical reports were used for establishing which of the neck spaces were involved by infection.

Also analyzed were the mortality rate and associated factors.

Data were tabulated for descriptive and statistical analysis using the Mann-Whitney test. The SPSS © version 10.0 software was used.

The Research Ethics Committee approved the project (protocol number 224/05).

## RESULTS

**Figure 1:**
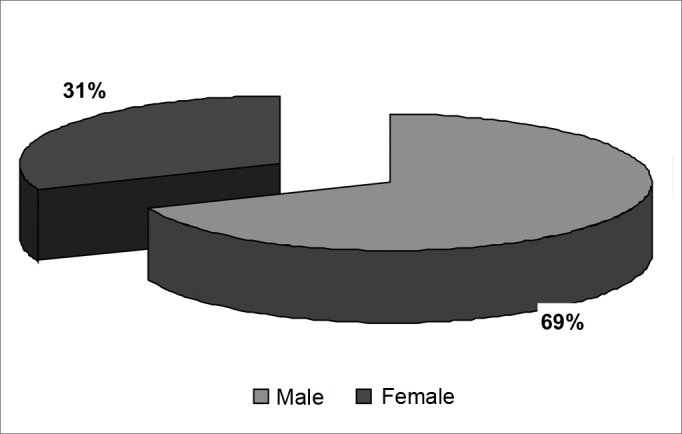
Distribution according to gender.

**Figure 2:**
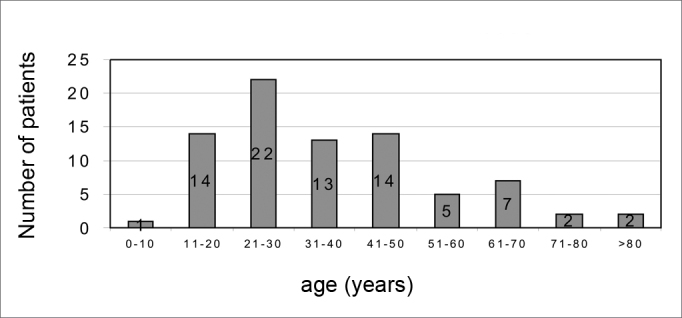
Distribution according to age.

**Figure 3:**
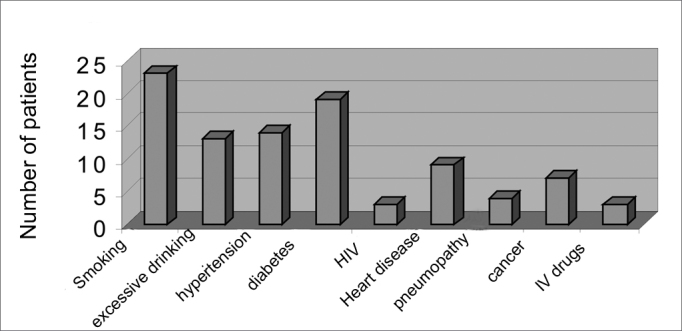
Habits and associated disorders.

There were 55 male patients and 25 female patients (Figure 1). The predominant race was white (66 patients), followed by black (7 patients) and brown (7 patients). Fourteen patients had arterial hypertension (17.5%), 19 were diabetic (23.75%), 9 were cardiopaths (11.25%), 4 had lung diseases (5%), 7 had malignant neoplasms (8.75%), 3 were HIV-positive (3.75%) and 3 were users of illegal drugs (3.75%). The mean age was 37.1 years (ranging from 2 months to 94 years) (Figure 2). Smoking was reported by 23 patients (28.75%) and use of alcoholic beverages was reported by 13 patients (16.25%) (Figure 3).

Odontogenic conditions were the most frequent causes of DNI (27.5%), followed by tonsillar diseases (22.5%), skin infection (8.75%) and parotid infection (6.25%). The cause was unclear in 20 patients (25%). Other causes (10%) were ganglionar tuberculosis with abscess (n=3), local trauma (n=2), complicated otitis media (n=1), infected thyroglossal cyst (n=1) and deep infection related to a central venous catheter (n=1). (Table 1)

**Table 1:** Causes of deep neck infections.

Cause	n	%
Odontogenic	22	27.5
Tonsillar	18	22.5
Skin infection	7	8.7
Parotid	5	6.2
Ganglionar tuberculosis	3	3 7
Trauma	2	2.5
Complicated otitis media	1	1.2
Infected thyroglossal cyst	1	1 2
Infected central catheter central	1	1.2
Unknown	20	25

**Figure 4:**
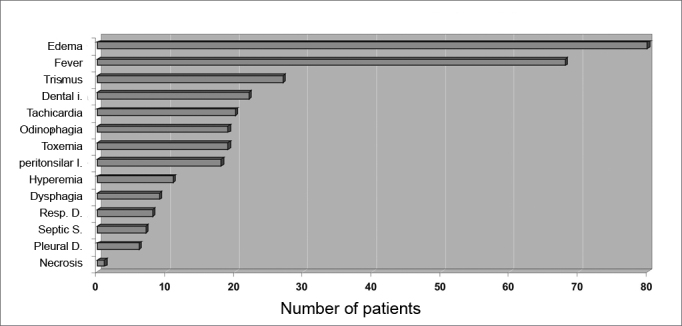
Clinical presentation of deep neck-facial disorders.

The symptoms of DNI were neck and/or facial edema in all patients, local pain in 79 patients (98.75%), fever in 68 patients (85%), odynophagia in 19 patients (23.75%), dysphagia in 9 patients (11.25%), difficult breathing in 8 patients (10%) and dental pain in 3 patients (3.75%). The physical examination demonstrated edema of the neck in all patients. There was dental infection in 22 patients (27.5%), peritonsillar infection in 18 patients (22.5%), trismus in 27 patients (33.75%), tachycardia in 20 patients (25%) and toxemia in 19 patients (23.75%). Septic shock was seen in 7 patients (8.75%). A pleural effusion was found in 6 patients (7.5%). Signs of skin necrosis were present in 1 patient. There was hyperemia in the furculum and thorax in 11 patients (13.75%) (Figure 4).

The mean progression time of DNI was 8.51 days; the mean hospital stay was 13.3 days.

Airways that were difficult to access occurred in 20 patients (25%); 12 of these patients required endoscopy for intubation, 3 underwent cricothyroidotomy upon admission, and 5 were intubated but considered difficult cases by anesthesiologists. There were no retrograde intubation cases.

Surgery was done in 78 patients, of which 55 patients underwent neck drainage only. Drainage of the neck with debridement of tissues was done in 7 patients; drainage of the neck and tracheostomy was done in 6 patients; drainage of the neck and thoracotomy was done in 5 patients; drainage of the neck and thoracic drainage was done in 4 patients; drainage of the neck and mastoidectomy was done in 1 patient. All of the patients that were drained received endovenous antibiotics. Conservative therapy with endovenous antibiotics and anti-inflammatory drugs was used in 2 patients (Table 2).

**Table 2:** Treatment of deep neck infection.

Treatment	n	%
Cervical drainage	55	68.75
Cervical drainage and debridement	7	8.75
Cervical drainage and tracheostomy	6	7.50
Cervical drainage and thoracotomy	5	6.25
Cervical and thoracic drainage	4	5.00
Cervical drainage and mastoidectomy	1	1.25
Conservative	2	2.50

Surgical findings were: pus in 64 patients, necrotizing fasciitis with pus in 9 patients, necrotizing fasciitis with no pus in 3 patients, and fasciitis with no necrosis in 2 patients.

The mean white blood count was 16,656 cells per cubic millimeter, ranging from 1,100 to 51,500 cells per cubic millimeter.

**Figure 5:**
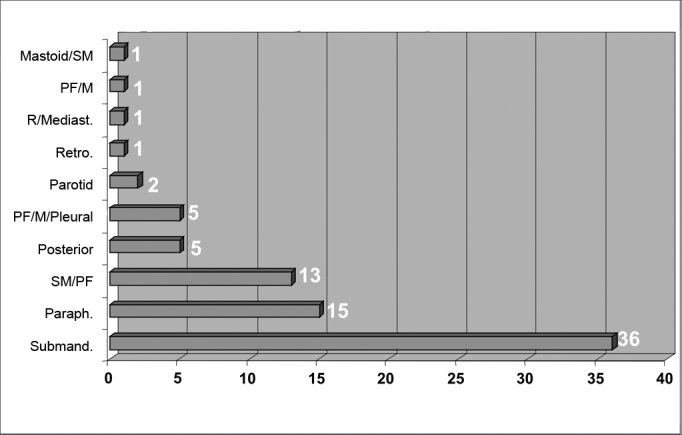
Neck-facial infection sites

Infection was located in the following neck spaces: the submandibulary space in 36 patients, the parapharyngeal and submandibulary spaces in 13 patients, the parapharyngeal space only in 15 patients, the posterior region of the neck in 5 patients, the parapharyngeal, mediastinal and pleural spaces in 5 patients, the parotid space in 2 patients, the retropharyngeal space in 1 patient, the retropharyngeal and mediastinal spaces in 1 patient, the parapharyngeal and mediastinal spaces in 1 patient, and the mastoid region and submandibulary space in 1 patient (Figure 5).

The infectious bacteria are shown on Table 3. There were 65 positive cultures. The most commonly isolated bacteria was Staphylococcus aureus in 30 patients (37.5%), followed by Group G Streptococci in 20 patients (25%). Thirteen cultures had no bacterial growth after 48 hours incubation.

**Table 3:** Bacteriology.

Microorganism	N	%
*Staphylococcus aureus*	30	37.50
*Streptococcus grupo G*	20	25.00
*Streptococcus viridans*	10	12.50
*Streptococcus pyogenes*	02	2.50
Bacterioides sp	01	1.25
*Pseudomonas aeruginosa*	01	1.25
*Klebisiela pneumoniae*	01	1.25
Peptostreptococcus species	01	1.25
*Prevotella melaninogenica*	01	1.25
Negative cultures	13	16.25

Ten patients had complications, shown on Table 4. Mediastinitis was the most severe complication. Only one of five patients with mediastinitis survived.

**Table 4:** Complications of deep neck and facial infections.

	N	%
Septic shock	7	8.75
Pleural effusion	6	7.50
Mediastinitis	5	6.25
Pericardial effusion	1	1.25
Upper airway obstruction	3	3.75
Gl hemorrhage	1	1.25
Necrosis of the skin	1	1.25

The mortality rate was 11.25% (n=9). Of the 9 deaths, 5 were male and 4 were female. Four were diabetics, 1 had bone marrow aplasia, 1 had cancer of the colon that was being treated by chemotherapy, and 1 had gastric cancer. The most common primary cause in these cases was tonsillar disease (n=4), followed by dental conditions (n=3); the origin was not defined in 2 cases. The sites of infection in these cases were: the parapharyngeal space in 8 patients and the retropharyngeal space in 1 patient. The upper mediastinum was involved in 3 of these cases, and the whole mediastinum was involved in 2 of these cases; in the latter there was unilateral pleural effusion. The patient that had involvement of the retropharyngeal space also had a large bilateral pleural effusion. Three patients were reoperated, requiring a second drainage of the neck (n=1) and thoracotomy (n=2). Seven patients died due to septicemia, 1 due to an acute myocardial infarction, and 1 due to a malfunctioning heart pacemaker.

The Mann-Whitney method was used for the statistical analysis to establish possible predisposing factors for a poor prognosis of this infection. The group that progressed to death (demise group) was compared with the group with a favorable outcome (non-demise group).

Clinical presentation, infection site, surgical findings and complication factors, as described above, were analyzed, taking into account a statistical significance of p<0.05.

In the clinical presentation, the presence of tachycardia (heart rate over 80 beat per minute), signs of toxemia, signs of tissue necrosis and respiratory failure were statistically significant factors in the demise group (Table 5).

**Table 5:** Analysis of potential factors of a poor prognosis of DNI in the clinical presentation.

	Demise grou (%)	Non-demise group (%)	p
Tachycardia	66.7	19.7	0.002
Toxemia	55.6	19.7	0.018
Tissue necrosis	11.1	0	0.005
Respiratory failure	55.6	4.2	<0.001

As to the infection site, involvement of the parapharyngeal space, the association of the parapharyngeal-mediastinal-pleural spaces, or the retropharynx-mediastinum was statistically significant in the demise group (Table 6).

**Table 6:** Analysis of the infection site.

	Demise group (%)	Non-demise group (%)	p
Parapharyngeal	55.6	14.1	0.003
Parapharyngeal + Medias tinal + Pleural space	22.2	4.2	0.037
Retroharyngeal + Mediastinal	11.1	0	0.005

In the surgical findings, only the presence of the association between fasciitis and pus was statistically significant in the demise group (Table 7).

**Table 7:** Analysis of surgical findings.

	Demise group (%)	Non-demise group (%)	p
Fasciitis with pus	55.6	5.6	<0.001
Fasciitis with no pus	11.1	2.8	0.220

Among the complications, the presence of septic shock and mediastinitis were statistically significant variables associated with the demise group (Table 8).

**Table 8:** Analysis of complications of DNI.

	Demise group (%)	Non-demise group (%)	p
Septic shock	55.6	2.8	<0.001
Mediastinitis	33.3	5.6	0.006

## DISCUSSION

Our results show interesting similarities and differences with the literature.

The mean age that was affected most by infection in the literature varied from 36 to 57 years,^2^^,^^3^^,^^5^, ^6^, ^7^, ^8^, ^9^, ^10^ which was similar to our findings. The disease was twice more frequent in males, as many authors have reported.^5^, ^6^, ^7^, ^8^^,^^11^

Smoking and drinking alcohol were the most commonly associated social habits. The most commonly found systemic diseases were diabetes and systemic arterial hypertension. The literature has reported a 16% to 20% incidence of diabetes.^8^ Systemic arterial hypertension, which may be associated with heart and lung diseases, is not given importance; these factors may have an influence on the morbidity and mortality of DNI. The HIV has been found in 7% of cases^6^ in the literature; in our study, however, it was found in only 3 patients (3.75%).

The clinical picture of infection with edema of the neck, odynophagia, fever, trismus, a poor health status, associated or not with a primary condition is similar to that found in the literature.^8^^,^^12^ In our study, the presence of tachycardia, toxemia, signs of tissue necrosis and respiratory failure were associated with a poor prognosis in these patients.

DNI originate from a variety of sites in the head and neck; these include the teeth, the salivary glands, lymphoid tissues and the tonsils. The teeth are the most common primary site (31% to 80%), followed by the tonsils (1.5% to 3.4%);^1^^,^^5^, ^6^, ^7^, ^8^^,^^10^, ^11^, ^12^, ^13^, ^14^, ^15^, ^16^, ^17^, ^18^ the latter is more frequent in children.^19^, ^20^, ^21^, ^22^ Odontogenic conditions were the most common cause in our series (27.5%), followed by tonsillar disease (22.5%). The cause remained unknown in 20 patients (25%), notwithstanding a detailed clinical history, physical examination and radiological studies. The oropharynx was probably the site of origin in these cases. Other studies have also shown a significant proportion (around 16% to 39%) of DNI of unknown origin.^6^, ^7^, ^8^, ^9^

About two thirds of the cultures of secretions were polymicrobial.6 The most commonly isolated organisms are mostly part of the normal oropharyngeal flora.^7^ In our series, Staphylococcus aureus was the most commonly isolated bacteria (37.5%), followed by Group G Streptococci (25%). Authors have presented a wide variation of bacteria associated with mixed infection; the most common bacteria that have been encountered have been Streptococci viridans, Staphylococcus epidermidis and Staphylococcus aureus.^4^^,^^6^^,^^8^ There was no bacterial growth in 13 cultures (16.25%); this is a low rate compared to that of other authors (which has ranged from 27% to 40%).^8^^,^^11^ This is probably due to the indiscriminate use of antibiotics prior to hospital admittance and the high doses of endovenous antibiotics before surgery.^8^

The most commonly involved areas in our study were the submandibulary and parapharyngeal spaces. Moncada et al.^23^ have established the dissemination routes for odontogenic neck infections; these authors demonstrated the anatomical relations between the submandibulary and parapharyngeal spaces, and have explained the pathophysiology of Ludwig's angina. Involvement of the parapharyngeal space, the association between the para-pharynx + mediastinum + pleura, or the retropharynx + mediastinum were associated with a poor prognosis.

Since the 1970s, computed tomography has evidently helped improve the diagnosis of DNI.^24^, ^25^, ^26^ Tomography of the neck and thorax establish the extension of infection and make it possible to precisely plan the treatment.

All of the patients in whom the abovementioned spaces were involved were treated surgically, except for 2 patients that had a well-defined peritonsillar infection in the suprahyoid area of the neck. These two patients had been using non-prescribed antibiotics for a prolonged period and had no signs of significant toxemia; they progressed well with high doses of endovenous antibiotics, and there was spontaneous intraoral drainage.

Special attention should be given to airway management when patients present trismus or signs of upper airway obstruction, particularly in Ludwig's angina, in which there is edema of the oral floor due to bilateral submandibulary space infection. Parhiscar et al.^6^ analyzed 210 patients with neck abscesses and reported a need for tracheostomy under anesthesia in 44% of cases, which demonstrates the severity of this condition. We agree that adequate management of airways with intubation using flexible fibroscopy and/or a tracheostomy in cases of significant trismus and edema of the tongue is a priority in the initial approach to DNI.^27^^,^^28^

Our complication rate was 12.5%, which is similar to findings in the literature; these have ranged from 12.85% to 25.5%,^6^^,^^9^^,^^11^ mostly associated with mortality. DNI are severe conditions that may rapidly progress to necrotizing fasciitis; descending mediastinitis, in which the mortality rate is 40% to 50%, may or may not complicate the condition, due to its rapid progression to septic shock. The mortality rate in our series was 11.25% (n=9); 7 patients died due to infection and 2 patients died due to non-infectious causes. Mediastinitis and septic shock were related to a poor prognosis of the infection.

Prompt recognition and treatment of DNI are essential for an improved prognosis. Thus, key elements for improved results are the identification of morbid factors, signs and symptoms, and computed tomography.

**Figure 6:**
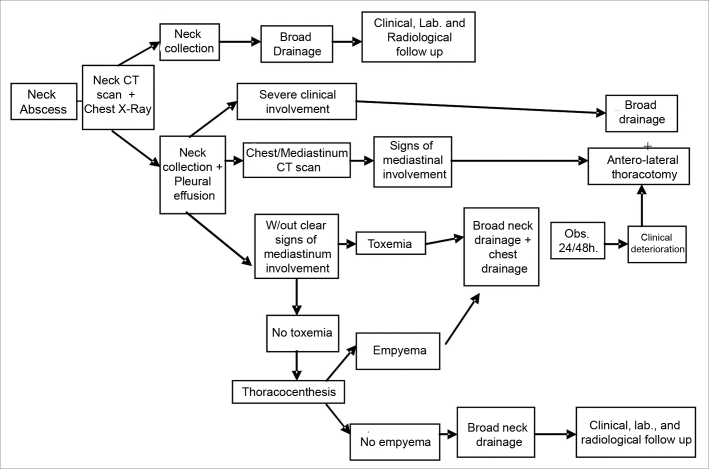
Treatment algorithm in Neck Infections

Based on our experience, we elaborated an algorithm (Figure 6) for managing DNI.

## CONCLUSIONS

The current study enabled us to conclude that:

1. Odontogenic and tonsillar causes are the most common;
2. The submandibular and the parapharyngeal areas are the most frequently involved spaces;
3. Staphylococcus aureus and Group G Streptococci are the main microorganisms involved in this condition;
4. A priority in the treatment of DNI should be an adequate management of airways, if surgery is indicated, no muscle relaxant should be given;
5. Surgical drainage is the standard treatment of DNI;
6. Computed tomography is the test of choice for the diagnosis of DNI;
7. DNI have high morbidity and mortality rates, particularly when associated with septic shock and mediastinitis.
8. The proposed clinical management algorithm enables an improved diagnostic and therapeutic approach.
